# Could inhibition of metalloproteinases be used to block the process of metastasis?

**DOI:** 10.1002/cbf.3730

**Published:** 2022-07-05

**Authors:** Jesús Alba, Ramiro Barcia, Javier Gutiérrez‐Berzal, Juan I. Ramos‐Martínez

**Affiliations:** ^1^ Histobiomol, Hospital POLUSA Lugo Spain; ^2^ Faculty of Sciences University of Santiago de Compostela Lugo Spain; ^3^ Department of Biochemistry and Molecular Biology, School of Veterinary University of Santiago de Compostela Lugo Spain

**Keywords:** cancer, epithelial–mesenchymal transition (EMT), extracellular matrix (ECM), matrix metalloproteinases (MMPs), metastasis

## Abstract

Metastasis is a multisequential process that allows tumor cells to migrate to tissues distant from the primary tumor. Only a small number of cells escape from the primary tumor; however, the metastases generated are responsible for more than 90% of cancer deaths. Many metastatic processes initially require the total or partial start‐up of a program for the transformation of tumor epithelial cells into mesenchymal cells (EMT). The launching of the EMT program is stimulated by cytokines and other elements produced by the diverse types of cells composing the tumor stroma. In parallel, a process of destabilization of the extracellular matrix (ECM) takes place by means of the synthesis of proteases of the matrix metalloproteinases (MMPs) family. EMC degradation allows the exportation of some tumor cells as mesenchymal cells to the circulatory system and their subsequent implantation in a tissue distant from the primary tumor. The blocking of these both processes appears as a hypothetical stop point in the metastatic mechanism. The present review deals with the different options to achieve the inhibition of MMPs, focusing on MMP7 as a target given its involvement in the metastatic processes of a wide variety of tumors.

## Significance statement

The simultaneous implantation of the epithelial–mesenchymal program and the synthesis and activation of matrix metalloproteinases during the first phases of the metastasis process is known. The inhibition of proteases could constitute a possible blockage of the process. The review describes the evolution of the different inhibition mechanisms that could inform applicable therapeutic mechanisms for the paralysis of the metastatic process.

## 1 INTRODUCTION

Cancer comprises more than 180 different diseases which are the result of a wide variety of complex molecular interactions. The systematization of common features started in 2000 with the publication of “*Hallmarks of Cancer*”^1^ allowed to identify the six characteristic features of a malignant tumor, among which the capacity to invade tissues and create metastasis stands out. In 2011, the same authors performed an update^2^ which included two new features.

Although the use of innovative technologies has led to an increased knowledge of the biological mechanisms that result in the formation of a primary tumor; however, the knowledge of the invasion‐metastasis mechanism is limited by the lack of specific technologies able to differentiate each of the different stages of the process.^3^

The capacity to invade the surrounding tissues and the spread of tumor cells to other organs, their settlement in those new tissues, and the formation of secondary tumor masses or metastasis is a multisequential process.^3^, ^4^, ^5^ All the properties gained initially by tumor cells combine to furnish capacities that enable them to abandon the primary tumor and settle in another tissue. In this sense, it can be said that seven of the eight features acquired serve to provide tumor cells with the properties that will enable their evasion and the subsequent formation of metastasis.^1^, ^2^ Regardless of the way in which tumor cells evade, individually or forming clusters, only a reduced number of cells will be able to complete the process and settle on tissues away from the primary tumor. This process is rather ineffective^3^; however, metastasizing of a tumor worsens notably the prognosis from the clinical point of view^6^; it is known that the metastasis process is responsible for 90% of deaths by cancer.^7^

The beginning of a metastatic process involves the activation of two apparently simultaneous mechanisms that have been the subject of study and interest. One of them refers to the transformation of tumor epithelial cells into a mesenchymal phenotype by the implantation of the molecular program termed epithelial–mesenchymal transition (EMT).^8^ Many authors suggest that the EMT program is a prerequisite within the metastatic mechanism.^9^, ^10^ The other process is the degradation of the tissue adjacent to the tumor by the destruction of the extracellular matrix (ECM), which enables the evasion of the tumor cells to the lymphatic system in search of the metastasizing spread.

## 2 THE EMT PROGRAM

The EMT program is not only involved in the initial stages of metastatic processes where, apparently, it could be a prerequisite for intravasation.^9^ Post EMT is also involved in embryogenesis, wound healing, fibrosis, and so on.^9^, ^10^, ^11^, ^12^, ^13^, ^14^, ^15^

**Figure 1:**
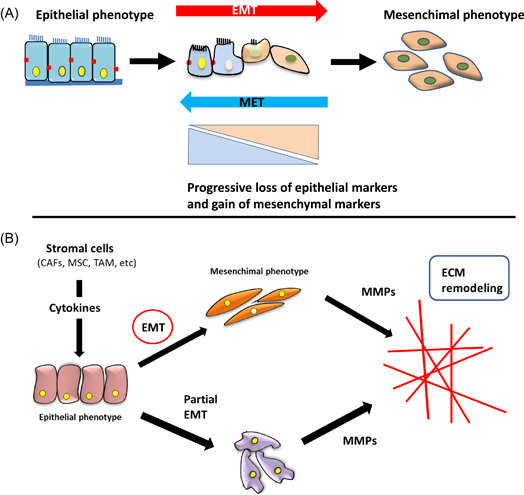
(A) Phases of the full or partial implementation of the EMT program. (B) Relationship of EMT program with MMPs synthesis and subsequent remodeling of the extracellular matrix (ECM). Stromal cells: Cancer‐associated fibroblasts (CAFs), mesenchymal stem cells (MSCs), and tumor‐associated macrophages (TAMs) release different cytokines that induce EMT implementation.^17^ Both stromal and tumor cells generate micro RNAs (miRNAs) that get involved in MMPs synthesis too. EMT, epithelial–mesenchymal transition; MMP, matrix metalloproteinase.

EMT is a process that leads neoplastic or normal epithelial cells to lose apico‐basal polarity, breaking the cell junctions (tight junctions, gap‐junction, and adherence‐junctions).^13^ EMT implementation is promoted by the action of peptides and molecular signals synthesized in an autocrine way and by the different cell elements present in the tumor stroma.^16^, ^17^ Environmental hypoxia coupled with the presence of cytokines and growth factors^13^, ^18^ induces the expression of different and specific transcription factors (EMT‐TF; (Twist, Snail, ZEB1 and ZEB2, and others) that provoke a progressive loss of expression of the epithelial phenotype markers, such as E‐cadherin, claudins, occludins, cytokeratins α6β4, integrins, and others.^19^, ^20^ Simultaneously, ETM‐TFs also induce the increase of the expression of mesenchymal markers, like N‐cadherin, vimentin, fibronectin, or β1 and β3 integrins (Figure 1).^10^, ^13^

In “in vitro” cultures, epithelial cells advance throughout the implementation of the EMT program turning progressively into a mesenchymal phenotype, losing the expression of E‐cadherin, main marker of the epithelial character.^21^ However, the cells subjected “in vivo” to an EMT program seldom finish the program with a full conversion into mesenchymal cells. In most cases, the program proceeds through cells with mixed phenotypes that keep part of the epithelial character and of the structure typical of the mesenchymal cells. This is called partial EMT state.^21^, ^22^ These cells adopt a migratory phenotype characterized by its capacity to resist immunosuppression and drugs^23^, ^24^, ^25^ and the evasion from apoptosis.^26^ It was also observed to favor the inflammation associated with cancer progression and fibrosis. In this sense, some evidence suggests that some cells under the EMT program acquire properties of tumor stem cells (GSC).^27^, ^28^ The fact that some epithelial characters are preserved permits the reversible process (mesenchymal–epithelial transformation) (MET) that starts after the tumor cell has settled, enabling metastasis formation and the gain of effectiveness.^15^, ^29^

## 3 CONSEQUENCES OF THE IMPLEMENTATION OF THE EMT PROGRAM

The EMT‐program implementation may induce the synthesis of different endo proteases that provoke ECM destabilization.^17^, ^30^, ^31^ In this sense epithelial tumor cells partially converted into mesenchymal cells contribute to ECM destruction. Also, secrete vascularization factors that favor the synthesis of capillaries that may serve as an escape way for metastatic cells.^32^ Angiogenesis is parallel to the development of the EMT program. It is a comprehensive program, because the production of vascular endothelial growth factor (VEGF) and EGF‐receptor (EGFR) is associated with the Twist2 pathway and to the reduction of the E‐cadherin expression.^33^, ^34^ Also, the hypoxia and acidity of the tumor environment produced by the accumulation of lactic acid (Warburg effect) adds up to ECM destabilization.^35^, ^36^

ECM and basal membrane degradations are critical for invasion and metastasis and account for a poor prognosis in many types of cancer.^37^ Simultaneously to the implementation of the EMT program, migration of the tumor cells occurs, favored by the production, secretion, and activation of the matrix metalloproteinases (MMPs).^38^, ^39^

## 4 MATRIX METALLOPROTEINASES

Excellent reviews have been published last years dealing with the structure and enzymatic activity of the different MMPs, which permitted their classification, and the knowledge of their specific cell location and substrates, an aspect that generates its own classification system.^40^, ^41^

In a basal situation, the presence and production of the different MMPs must be perfectly regulated, because the excess activity may cause cellular chaos.^30^, ^42^ In a natural way, the presence of the different MMPs is strictly regulated at various levels; in this sense, MMPs synthesis is modulated at transcription and transduction levels and by post‐transductional modifications.^41^ Like nearly other peptidases, MMPs are synthesized as inactive pro‐enzymes and are activated by proteolysis when losing the peptidic element in the amino‐terminal end.^43^ In their active center, MMPs have a highly preserved sequence next to the C‐terminal called “*cysteine switch*,” where cysteine residues bind in the catalytic site through Zn^2+^, transforming MMP into a latent protein. The rupture of the linkage means the pro‐domain loss and MMPs activation.^44^ Full activation is achieved through autocatalysis or by the mutual and specific intervention of other MMPs.^30^, ^45^

MMPs proteolytic activity is controlled by four types of MMPs inhibiting proteins (TIMPs) that bind the catalytic site in a specific and reversible mode.^46^, ^47^, ^48^ Other peptides like α2‐macroglobulin or thrombopodin1 can also inhibit the MMPs, but less effectively.^49^

The overexpression of the different MMPs is well documented for many types of tumors,^50^, ^51^, ^52^ with a positive correlation between tumor aggressiveness and protease expression.^53^ The diverse cells composing tumor stroma secrete specifically the different MMPs; however, many tumor cells express MMP7, apparently in parallel with the implementation of EMT program.^54^ The rest of cells present in the tumor stroma produce diverse MMPs that interact with each other in a complex net of proteolytic processes that seem to converge in the synthesis of MMP7, MMP2, and MMP9.^45^, ^53^, ^55^

**Figure 2:**
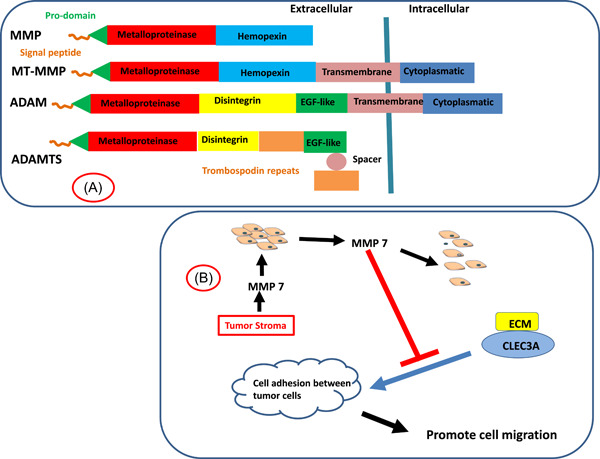
(A) General structure of MMP family and MMP7. (B) Effect of MMP7 on ECM: MMP7 enhances the fibroblast isolation process through a direct action on the C‐type lectin domain family 3 member A (CLEC3A) on the tumor surface, destabilizing the adhesive activity of the cells.^57^ ECM, extracellular matrix; MMP, matrix metalloproteinase.

MMP7 is the smallest matrix metalloproteinase with a molecular weight of 27 kDa in its latent form and 19 kDa in its active form. This suggests that it might be an end‐protein that is the result of many of the MMPs‐activating mechanisms. In this sense, MMP7 is the most specific of all MMPs with respect to target substrate, with a particularly mighty action on MMP2 and MMP9.^45^, ^56^ MMP7, also known as Matrilysin, is a Zinc‐dependent endopeptidase consisting of 267 amino acids (Figure 2A). Unlike other MMPs, MMP7 lacks the Hemopexin domain in the C‐terminal, and MMP7 expression is regulated by the Wnt/β‐catenin pathway and can be stimulated by TFG**‐**β.^58^, ^59^ The implementation of EMT program by tumor cells and MMP7 synthesis share the Wnt/β‐catenin pathway, as proved by the simultaneous inhibition of the phenotype transformation and the inhibition of MMP7 direct production by hydroxysteroid sulfotransferase 2B1b (SULT2B1b) or Thymoquinone.^60^, ^61^, ^62^

The increase of MMP7 expression in diverse types of tumors has been shown in many studies. MMP7 can be considered a biomarker in tumors of the digestive tract like colon,^63^, ^64^ pancreas,^65^, ^66^ or gastric cancer.^67^ It also showed its utility in different tumors on breast cancer,^68^ urogenital cancer,^69^, ^70^ small cell lung cancer,^71^ or melanoma.^72^ In all cases, MMP7 appeared as a necessary element in invasion and metastasis mechanisms and may be considered not only a diagnostic biomarker for a variety of tumors, but also a marker of poor prognosis. This last suggestion can extend to postsurgical situations.^73^

These results suggest that a setting capable of modulating the presence of MMP7 could be considered a therapeutic target, where the inhibition of the activity, the cancellation of the synthesis, or the expression of the protease could block metastasis process (Figure 2B).

## 5 MMPs INHIBITION SYSTEMS

As previously commented, MMPs activities are naturally modulated by the presence of a protein family known as tissue inhibitors of MMPs (TIMPs). In human tissues, four types of TIMPs with a wide MMPs inhibitory spectrum were detected.^46^, ^74^ TIMP gets into the active center, blocking the catalytic Zn^2+^ and removing the water bound to the cation. It is a classic natural chelation mechanism that blocks the proteolytic action of the different MMPs.^41^, ^47^, ^75^, ^76^

Other strategies consisting in the chelation of the Zn^2+^ atom present in MMPs active center served to design different chemicals that might inhibit different MMPs, such as molecules with hydroxamic acids, carboxylates, thiols, or phosphonic acids with the ability to block the endo protease activity. The highest inhibiting potential was obtained with agents containing hydroxamate and carboxylate‐based groups (Marimastat, Batimastat, and Ilomastat); however, the inhibiting potential is little specific in the blocking of the different MMPs, as happens with TIMPs. The cause is the structural similarity of the active centers of the different endopeptidases. Implemented clinical trials, some reaching level III, have been abandoned due to their adverse musculoskeletal side effects and poor oral bioavailability, but not due to their inhibiting effectiveness.^30^, ^77^

The use of anti‐MMPs specific antibodies was proposed as an alternative to Zn^2+^‐chelating agents and the blocking of MMPs active center by TIMPs. The few studies performed so far confirm a reduced specificity of the monoclonal antibodies used against MMPs antigenic elements that show little variability.^77^, ^78^

The simultaneity in the implementation of the EMT program and the synthesis of MMPs requires the activation of transcription factors that may be related or pass through common pathways. One strategy arises in the search or design of inhibitory molecules of one of the two programs that can also affect the other. It has been previously described that both situations share upstream the activation of the pathway Wnt/β‐catenin. In this sense, thymoquinone cancels the invasion and metastasis in bladder cancer through the inhibition of Wnt/β‐catenin.^63^ Inhibition of the same pathway by cinobufacine and XAV939 is detected in colon cancer.^66^, ^79^ Besides the implication of Wnt/β‐catenin, apatinib appears as a potent inhibitor of invasion and metastasis in liver cancer by reducing the flow through NF_k_B signaling,^80^ a mechanism collateral to Wnt/β‐catenin. These results are like those obtained with celastrol, which inhibits PI3/AKT signaling pathway,^64^ another pathway adjacent to Wnt/β‐catenin.

Many microRNAs (miRNAs) and long non‐coding RNAs (lncRNAs) have been related to cancer, and its aberrant expression has been detected in solid tumors and in hematological malignancies.^81^, ^82^, ^83^ Many of miRNAs or lncRNAs genes are located in genomic regions associated with cancer or in fragile sites, which suggests an important role in the disease evolution.^84^, ^85^ In this sense, in recent years, the two types of RNAs have emerged as extremely useful in disease diagnosis.^80^, ^86^ Moreover, some of the miRNAs or lncRNAs studied are highly cross‐cutting, because their hyperproduction, and in some cases their use can involve different tumor types, as happens with miRNA‐21.^87^, ^88^, ^89^, ^90^

Some miRNAs or lncRNAs have a bifacial character because in addition to being shown as markers of malignancy in many tumors, some can block the action of the counterpart. This last action sheds light on the possibility of being used as blocking agents in the expression of mRNAs, and therefore of tumor processes. The actions can be through three different mechanisms: one would consist of the blockade exerted by a specific miRNA on a lncRNA responsible for tumor progress; another could be the use of a specific lncRNA as a reservoir of miRNAs and the third mechanism would involve a competition between lncRNA and miRNAs to bind to the target mRNA.^91^, ^92^, ^93^ The use of miRNAs as blocking systems has been studied in “in vitro” assays with different tumors. Among the stages of cancer development, the involvement of miRNA as therapeutic remedy has been more clearly observed during the EMT implementation program and in the interruption of different functions of MMPs and ECM remodeling.^87^, ^94^, ^95^ In this sense, it has been detected the inhibition of EFGR and PI3K by miRNA‐34a that are lateral pathways to Wnt/β‐catenin.^96^, ^97^, ^98^ Gastric tumor cells reduce drastically their invasion capacity when treated with miRNA‐335‐5p, showing a remarkable decrease in MMP7 levels.^99^, ^100^, ^101^ In hepatocellular cancer, there is a direct action of miRNA‐298 upon the transcription of elements of the Wnt/β‐catenin pathway, whose consequence is the full cancellation of evasion and metastasis.^102^ The overexpression of miRNA‐335 causes the inhibition of migration and metastasis in osteosarcoma reducing MMP7 synthesis and EMT‐TF cancellation by inhibition of SN1P1.^103^ Similar results are obtained by different miRNA in breast cancer,^104^ esophageal cancer^105^ or melanoma.^106^

Most of the results mentioned above were obtained in tumor cell cultures and many of them have been confirmed in mouse models. Regardless of the action on the metastatic process, other miRNA families seem to verify similar inhibiting effects upon other tumor process triggers, such as the action of miRNAs‐34 or miRNA‐200, that act on tumor suppressors.^86^, ^107^

The delivery of miRNA to the specific tumor cell is resolved when its action is studied in cell cultures; however, study in whole animals or in human medicine requires the correct identification of the target. The first miRNA clinical trial on human patients used MRX34, a liposome containing miRNA‐34 for patients with advanced liver cancer. MRX34 is an RNA double helix identical to miRNA‐34 encapsulated in a liposomal nanoparticle.^86^, ^108^ In this sense, the recent advances developed with the vaccines against SARS‐COV‐2 have shed much light.

In the future, this technology can be assayed as the application of blockage processes in the implementation of metastasis.^57^, ^109^, ^110^, ^111^

## 6 CONCLUSIONS

From the present review, it is concluded that the processes of starting the EMT program and the synthesis of MMPs are simultaneous in some types of cancer and are the necessary requirement at the beginning of the metastasis mechanism. In many types of tumors, both processes share common signaling pathways, so an inhibitory process could affect the parallel. The inhibition of the synthesis of MPP7 may be an objective that would make it possible to control the metastatic phase of the disease. The review provides data on different or probable strategies to be carried out.

## AUTHOR CONTRIBUTIONS

All the authors have contributed in a similar way to the elaboration of the article. All authors have read and approved the final manuscript.

## CONFLICT OF INTEREST

The authors declare no conflict of interest.

## Data Availability

All data generated or analyzed during this study are included in the published article and references.
